# Intrinsically superparamagnetic Fe-hydroxyapatite nanoparticles positively influence osteoblast-like cell behaviour

**DOI:** 10.1186/1477-3155-10-32

**Published:** 2012-07-24

**Authors:** Silvia Panseri, Carla Cunha, Teresa D’Alessandro, Monica Sandri, Gianluca Giavaresi, Maurilio Marcacci, Clark T Hung, Anna Tampieri

**Affiliations:** 1 Laboratory of Biomechanics and Technology Innovation, Rizzoli Orthopaedic Institute, Bologna, Italy; 2 Institute of Science and Technology for Ceramics, National Research Council, Faenza, RA, Italy; 3 Laboratory of Preclinical and Surgical Studies, Rizzoli Orthopaedic Institute, Bologna, Italy; 4 Laboratory of Biocompatibility, Innovative Technologies and Advanced Therapies, Rizzoli Orthopaedic Institute, Bologna, Italy; 5 Department of Biomedical Engineering, Columbia University, New York, USA

**Keywords:** Nanoparticles, Superparamagnetism, Hydroxyapatite, Static magnetic field, Orthopaedic applications

## Abstract

**Background:**

Superparamagnetic nanoparticles (MNPs) have been progressively explored for their potential in biomedical applications and in particular as a contrast agent for diagnostic imaging, for magnetic drug delivery and more recently for tissue engineering applications. Considering the importance of having safe MNPs for such applications, and the essential role of iron in bone remodelling, this study developed and analysed novel biocompatible and bioreabsorbable superparamagnetic nanoparticles, that avoid the use of poorly tolerated magnetite based nanoparticles, for bone tissue engineering applications.

**Results:**

MNPs were obtained by doping hydroxyapatite (HA) with Fe ions, by directly substituting Fe^2+^ and Fe^3+^ into the HA structure yielding superparamagnetic bioactive phase. In the current study, we have investigated the effects of increasing concentrations (2000 μg/ml; 1000 μg/ml; 500 μg/ml; 200 μg/ml) of FeHA MNPs *in vitro* using Saos-2 human osteoblast-like cells cultured for 1, 3 and 7 days with and without the exposure to a static magnetic field of 320 mT. Results demonstrated not only a comparable osteoblast viability and morphology, but increased in cell proliferation, when compared to a commercially available Ha nanoparticles, even with the highest dose used. Furthermore, FeHA MNPs exposure to the static magnetic field resulted in a significant increase in cell proliferation throughout the experimental period, and higher osteoblast activity.

*In vivo* preliminary results demonstrated good biocompatibility of FeHA superparamagnetic material four weeks after implantation into a critical size lesion of the rabbit condyle.

**Conclusions:**

The results of the current study suggest that these novel FeHA MNPs may be particularly relevant for strategies of bone tissue regeneration and open new perspectives for the application of a static magnetic field in a clinical setting of bone replacement, either for diagnostic imaging or magnetic drug delivery.

## Background

Bioactive materials are currently at the cutting edge of regenerative medicine research due to the foreseeable need for bone tissue regeneration as an effective way to improve the current medical practice of bone replacement.

To regenerate bone tissue, the body relies on materials that it uses like a template to regenerate tissue [1]. The body is very capable of healing and regenerating itself when the defects are small. However, larger defects cannot be healed without making use of an aid, such as the employment of biomaterials. There are multiple criteria to design materials for bone regeneration, which include: osteoinductivity (capable of promoting the differentiation of progenitor cells down an osteoblastic lineage), osteoconductivity (support bone growth and encourage the ingrowth of surrounding bone), and osteointegration (integrative to the surrounding bone), biocompatibility (induce minimal toxic or immune response), safe and effective resorbability, similar mechanical properties to bone (so as to perform its load-bearing function), ability to shape to a wide range of defect geometries, and finally must meet all regulatory requirements for clinical use [1,2].

In the last decades, nanotechnology has been progressively used to enhance the above-mentioned bone tissue engineering strategies [3]. In particular, nanotechnology has been employed to overcome some of the current limitations associated with bone regeneration methods, including insufficient mechanical strength of scaffold materials, ineffective cell growth and osteogenic differentiation at the defect site [4]. In fact, decreasing the material size into the nanoscale, the surface area, the surface roughness and the surface area to volume ratios are dramatically increased leading to superior materials physiochemical properties and mimicking the hierarchical nanostructure of native tissue [5,6].

Moreover, recently, the usage of superparamagnetic nanoparticles (MNPs) for biological and medical purposes has been increasing [7-9]. MNPs contain a magnetic core (usually composed of magnetite Fe_3_O_4_ or maghemite γ-Fe_2_O_3_) that confers the unique feature of reacting to magnetic forces. This core is usually coated with an inert layer that minimises hydrophobic interactions, enhancing colloid dispersion and biocompatibility. MNPs have been used in the last decade for *in vitro* and *in vivo* applications, as hyperthermia [10], contrast agent for diagnostic imaging [11,12], magnetic drug delivery [13], and cell mechanosensitive receptor manipulation to induce cell differentiation [14]. Whereas only few authors proposed approaches for tissue engineering and in particular its use in orthopaedic applications remains largely uninvestigated to date [10,15,16]. This is despite the promise of iron to increase bone health. In fact the beneficial link between iron and bone density has been demonstrated in clinic by the association of dietary iron and a healthy bone mineral density [17,18].

Although the role of iron in bone accrual has received little attention, a few studies have previously shown that iron restriction can have an inhibitory effect on the mineralization of osteoblasts *in vitro* and experimental evidence also suggests that there may be some positive association between iron metabolism and the *in vitro* proliferation of bone or non-bone cell lines [19-21].

Considering the importance of having non-toxic MNPs for the above-mentioned applications, and the important role of iron in bone regeneration and remodelling, this study aimed to analyse for the first time novel superparamagnetic bioactive and bioresorbable nanoparticles obtained by doping hydroxyapatite (HA) with Fe ions in ideal condition aimed at limiting the formation of poorly tolerated magnetic secondary phase (i.e. Fe_3_O_4_) and able to be manipulated *in situ* by magnetic forces [22]. In fact, the use of magnetic stimulation in the field of regenerative medicine is emerging as one of the most attractive concepts [23-25].

In this context, the present study investigated the *in vitro* biocompatibility and bioactivity of FeHA nanoparticles in cultures of osteoblast-like cells in the absence and presence of an externally applied static magnetic field. These studies were followed subsequently by a pilot study to verify *in vivo* biocompatiability of these innovative FeHA materials.

## Results and discussions

Superparamagnetic FeHA nanoparticles have been synthesized following a neutralization method in which both Fe species are simultaneously introduced under controlled synthesis conditions. Following this synthesis method, during the stage of HA formation, the crystallographic position Ca(1) and Ca(2) of the apatite lattice are selectively substituted by iron species, Fe^3+^ and Fe^2+^ respectively [26] generating two distinct interacting structural domains whose interaction provide an intrinsic superparamagnetism [22]. The *in vitro* study was performed using human osteoblast-like cells firstly to evaluate any effect of novel FeHA nanoparticles on cell culture compared to HA nanoparticles already commercialized and used in the clinic as bone filler or as the primary component of several bone substitutes. Subsequently our attention was focused on the effect of static magnetic field on FeHA MNPs in cell culture. After obtaining positive and encouraging *in vitro* results, this magnetic biomaterial was tested for the first time *in vivo* in a pilot experiment in a rabbit condyle critical bone defect.

### Superparamagnetic FeHA nanoparticles characterization

Inductively coupled plasma (ICP) analysis evaluating the Fe, Ca and PO_4_ ions concentration, showed evidences of the replacement of Ca with Fe. In fact (Fe + Ca)/P molar ratio was very close to the theoretical one (Ca/P = 1.67) while the Ca/P = 1.41 molar ratio was lower than the theoretical one confirming the replacement of Ca with Fe ions [22].

**Figure 1:**
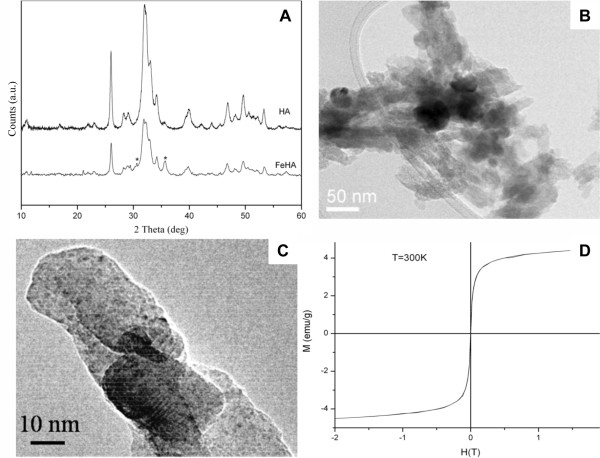
**FeHA nanoparticles characterization. A)** XRD analyses profiles obtained from FeHA and HA nanopowders synthesized at the same temperature and conditions. FeHA spectra underline a lower crystallinity and a little amount of magnetite (*) (~ 2 vol.%) as secondary phase. **B,C)** TEM micrographs showing FeHA MNPs with needle-like morphology and the absence of Fe agglomerates inside the particles. **D)** Magnetization curve obtained from the analysis of FeHA MNPs, which displays a typical superparamagnetic behaviour.

As detected by X-ray powder diffraction (XRD) analysis (Figure 1), the synthesis method employed leads to a low crystalline apatite with a crystallinity extent much lower than the non-substituted HA prepared at the same temperature with very low content of magnetite (<2 vol.%) as secondary phase. Moreover, XRD structural analysis also gave the evidence of the substitution of Fe ions into the HA lattice (Figure 1A): computer simulations clearly indicate that the Fe^2+^ and Fe^3+^ both occupy different Ca^2+^ positions in the HA lattice so that Fe ions are not situated in cell interstitial position but in Ca-substituting position.

Moreover, by Rietveld analysis an increase of the *a* axis from 9.4218(5) to 9.4557(1) and a decrease of *c* axis from 6.8813(3) to 6.8785(1) was detected as expected in the case of Ca substitution with ion species having a lower radius.

FeHA nanopowder morphology was investigated using TEM analysis that showed a very low concentration of dark spots (5-10 nm in size) corresponding to inclusions of iron rich phases (Figure 1B, C). In addition TEM investigation confirmed that the quasi-amorphous calcium phosphate matrix contains iron uniformly distributed and showed calcium phosphate particles with needle-like morphology, rather heterogeneous in size, 5-20 nm in width and up to 50-80 nm in length (Figure 1B, C).

For FeHA nanopowders the magnetization curve as function of the applied magnetic field showed the typical superparamagnetic behaviour of single-domain magnetic nanoparticles (Figure 1D). Contrary to what would be expected on the basis of the amount and aggregate size of magnetite present in the powder as secondary phase and detected by XRD, the saturation magnetization value of the FeHA nanopowder was higher (4.0 - 4.2 emu/g) confirming the intrinsic magnetic property of the powder due to the substitution of iron ions in the HA lattice [22].

### *In vitro* evaluation of biocompatibility and cell morphology

*In vitro* evaluation of the effects of the novel FeHA MNPs on osteoblast-like cell culture was conducted. In detail, 24 hours after Saos-2 cells were plated, 4 different concentrations of FeHA and HA nanoparticles, in a range from 200 μg/ml up to 2000 μg/ml, were added to the culture medium. It should be noted that the concentrations used in this study were significantly higher than the normal concentration used in several nanoparticles studies, in fact even the lowest concentration (200 μg/ml) is in many cases nearly the highest concentration adopted by several groups [21,27,28]. In this regard, the aim of this study was to verify any toxic effects induced by the FeHA MNPs compared to the HA nanoparticles, keeping in mind future *in vivo* applications, where high nanoparticle accumulation in the cells could influence cell behaviour.

**Figure 2:**
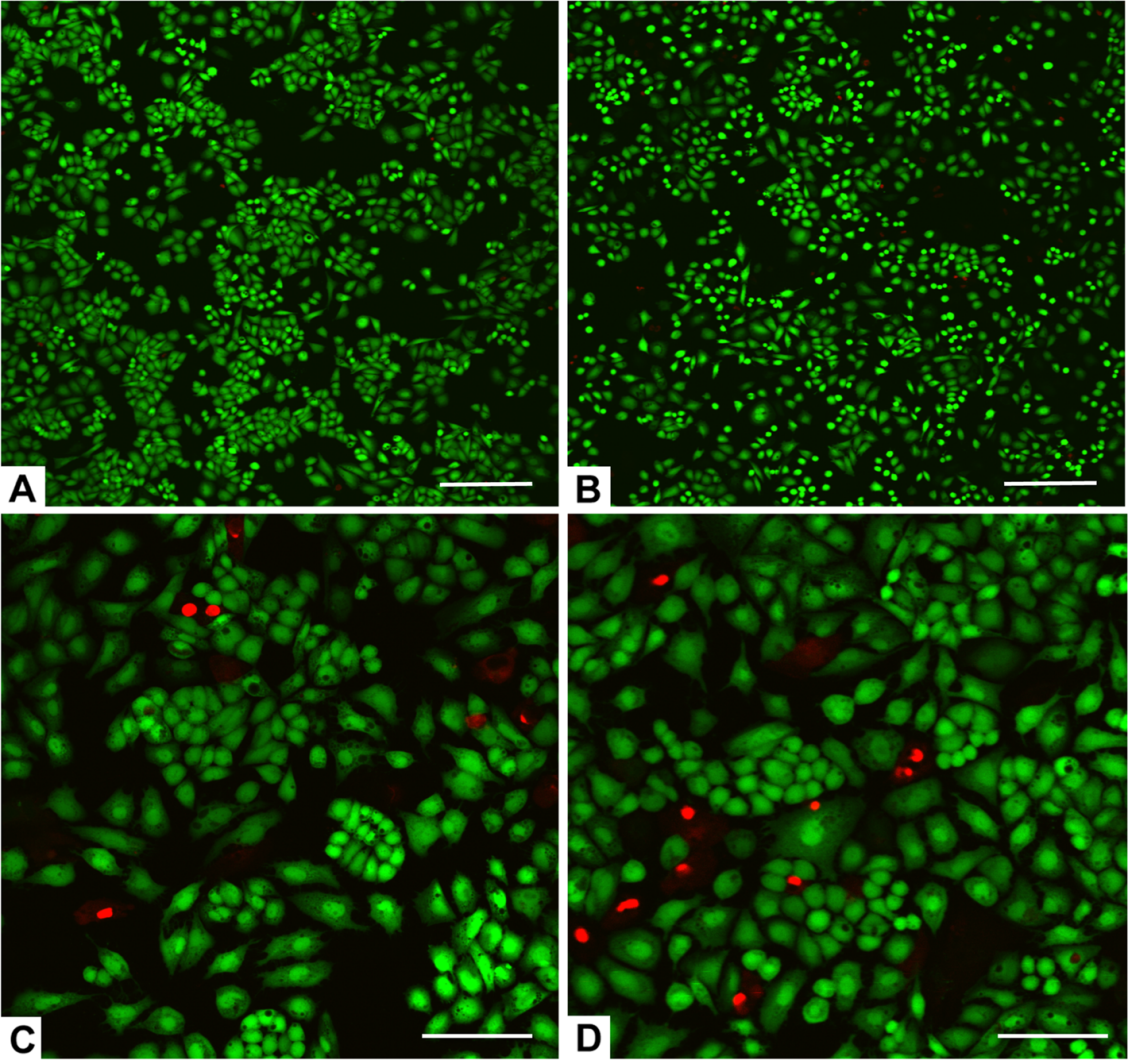
**Analysis of cell viability.** Cell viability was analysed by the Live/Dead assay (n = 2). Calcein AM stains for live cells in green, EthD-1 stains for dead cells in red. **A)** HA 1000 μg/ml at day 3. **B)** FeHA 1000 μg/ml at day 3. **C)** HA 200 μg/ml at day 7. **D)** FeHA 200 μg/ml at day 7. Scale bars: A,B) 200 μm. C,D) 100 μm.

**Figure 3:**
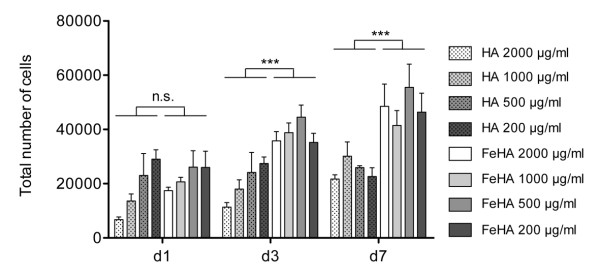
**Cell proliferation assay.** The Picogreen DNA content assay was performed on cultures of osteoblast-like cells seeded with 4 different concentrations of HA and FeHA nanoparticles at 1, 3 and 7 days of culture (n = 5). n.s. = not significant; *** p≤0.001.

The cell culture was analysed for 7 days for cell viability, with the Live/Dead assay showing for both FeHA and HA groups a very high ratio of viable cells at each experimental time point with no significant differences among the two groups, with a range respectively between 98.0±0.5% and 100% at day 1, 96.7±0.4% and 99.1±0.5% at day 3, 97.3±0.5% and 99.7±0.2% at day 7 (Figure 2). Furthermore, the presence of FeHA MNPs in the cell culture media positively influenced cell proliferation compared to HA nanoparticles. In fact, analysing the DNA content, significant differences were seen at day 3 and day 7 FeHA MNPs group and HA group (Figure 3). While at day 1 there was no statistic significant difference among the superparamagnetic and the control nanoparticles, there was a trend showing a higher number of cells in FeHA groups compared to the HA groups. These data are in agreement with several studies where different types of magnetic particles were used and they influence cell proliferation [21,28,29].

**Figure 4:**
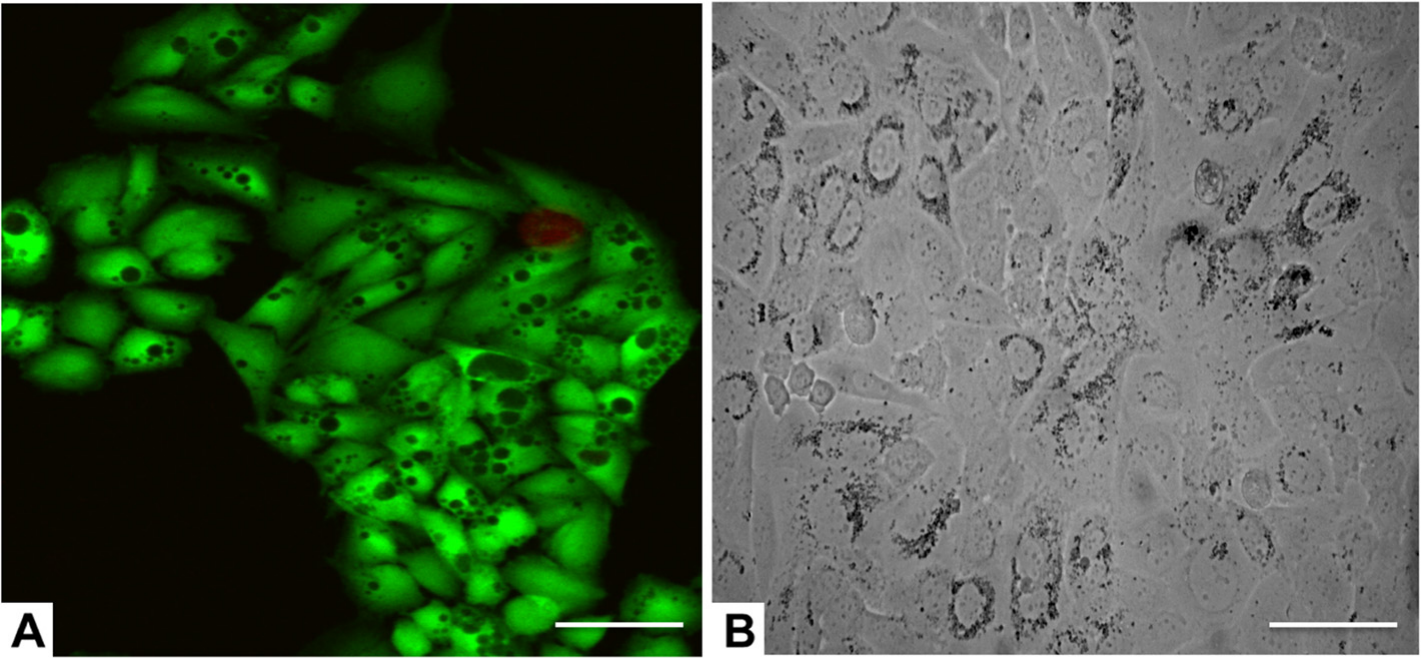
**Analysis of cell morphology.** Cells were spread with good morphology and firmly attached to the surface. Black spots are the MNPs that have been internalized by cells. **A)** FeHA 500 μg/ml at day 3, cells stained with Calcein AM in green and EthD-1 in red. **B)** FeHA 200 μg/ml at day 7, bright field image. Scale bars: 50 μm.

The obtained results demonstrate the good biocompatibility of the FeHA MNPs. In fact not only did they not reduce the cell viability (at even with the highest FeHA MNPs concentration), they also enhanced cell proliferation compared to respective HA groups. Looking in detail at the cell morphology, nanoparticles were seen accumulated in the cytoplasm (Figure 4A,B) and even when the cells showed high MNP accumulation they remained firmly attached to the well surface. This fact suggests that the novel intrinsically magnetic FeHA MNPs were well tolerated by the cells in a similar way as the HA groups, where HA serves as the main component of several bone substitutes already used in clinical applications, and have also been shown to induce cell proliferation.

As previously stated, we envisage a prominent role of FeHA MNPs will be in biomedical applications, particularly in conjunction with an applied magnetic field. Due to their superparamagnetic properties, the FeHA MNPs become magnetized upon exposure to a magnetic field without showing permanent magnetization (remanence) once the field is turned off.

### Effect of the *in vitro* application of a static magnetic field

As we have demonstrated similar biocompatibility of FeHA MNPs to HA particles, we focused our attention on studies designed to study how the presence of an externally applied static magnetic field (SMF) could modulate the cell proliferative effects and bone regenerative capacity induced by these MNPs. All the experiments were conducted with or without applying a 320 mT SMF on cells seeded with the 4 different concentrations of FeHA MNPs.

**Figure 5:**
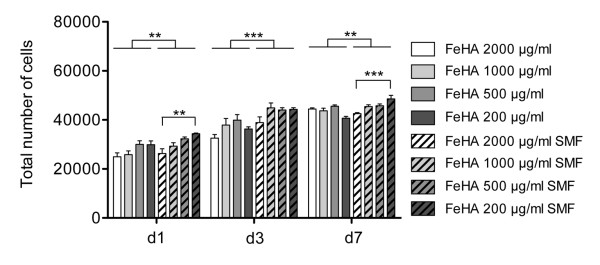
**Cell proliferation assay in the presence of a static magnetic field.** The Picogreen DNA content assay was performed on cultures of osteoblast-like cells seeded with 4 different concentrations of FeHA MNPs at 1, 3 and 7 days of culture, either in the presence or absence of a static magnetic field (SMF) (n = 5). ** p≤0.01; *** p≤0.001.

In the literature, there have been far fewer studies on the cellular effects of static magnetic field at the cellular level, compared to those of extremely low frequency electromagnetic fields. While several studies showed that exposure to static magnetic fields alone has no or extremely small effects on cell growth and genetic toxicity regardless of the magnetic density, in combination with other external factors such as ionizing radiation and some chemicals, there is evidence to strongly suggest that a SMF modifies their effects [30]. Our findings are strongly in agreement with these data. In fact, after confirming that SMF on cells in culture by itself did not affect cell behaviour (data not shown), our study with FeHA MNPs have confirmed a statistically significant increase in cell proliferation from day 1 to day 7 between FeHA groups with SMF application compared to groups without magnetic field application (Figure 5). In detail, the 200 μg/ml FeHA MNPs concentration induce a higher cell proliferation compared to the 2000 μg/ml FeHA MNPs only with exposure to the SMF, suggesting that the lowest concentration of nanoparticles acts synergy with the magnetic field to stimulate cell proliferation (Figure 5).

**Figure 6:**
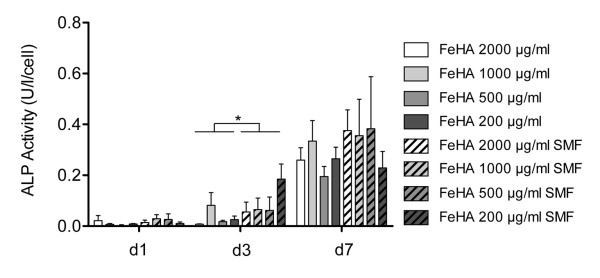
**Alkaline phosphatase activity assay in the presence of a static magnetic field.** AP activity was measured with different concentrations of FeHA MNPs seeded with human osteoblast-like cells at 1, 3 and 7 days, either in the presence or absence of a static magnetic field (SMF) (n = 5). * p≤0.05.

Furthermore, with respect to osteoblast activity on each condition, AP activity seemed to be influenced by the synergic effect of FeHA MNPs and SMF, with a significant effect at day 3, indicating that there might be also a positive effect on osteoblast activity given by the presence of SMF (Figure 6).

### *In vivo* pilot experiment

In order to assess how well our *in vitro* observations translate *in vivo,* we tested the biocompatibility of FeHA materials in a pilot animal study of bone repair (a rabbit critical bone defect model). For this purpose FeHA nanopowder was processed in order to obtain a granulate (400-600 μm) more easily handled and similar to bone fillers already used in clinical applications. The FeHA granules were not stabilized with the same thermal treatment applied to the reference material to avoid chemical-physical modification of the nanostructure and loss of their superparamagnetic features (see “Methods” section).

**Figure 7:**
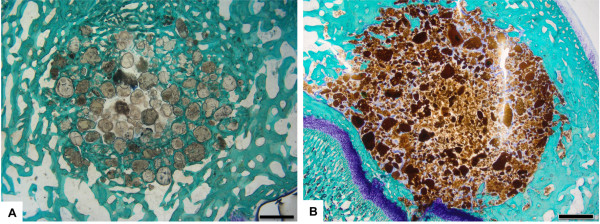
**Histological evaluation of the*in vivo*implanted HA and FeHA granules.** Toluidine Blue, Acid Fucsin and Fast Green staining shows similar histocompatibility for both biomaterials 4 weeks after implantation (n = 6). **A)** HA control group, **B)** FeHA group. Scale bars: 1 mm.

At 4 weeks post-implantation, macroscopic evaluation showed the HA and FeHA biomaterials to be in the proper position and there was no evidence of haematoma, edema, infection or tissue necrosis in either bone and peri-implant soft tissue associated with control or magnetic implants. Bone tissue was well visible around the biomaterials in both groups demonstrating a good bone integration of FeHA granules (Figure 7). In detail, between the granules, the magnetic FeHA group showed more immature bone not yet completely mineralized stained with Toluidine Blue respect to HA granulate control group (Figure 7). We speculate that this is probably due to the lower physical stability of the FeHA granules in comparison with the reference material, which has been subjected to the thermal treatment. This would result in a less compact material with a higher specific surface available for bone integration, possibly making FeHA more bioactive than control. In the long term these characteristics might positively influence tissue regeneration and stability and reduce biomaterial resorbability.

In any case, the aim of this *in vivo* study was merely to verify the histocompatibility of these new magnetic FeHA particles that for the first time were implanted *in vivo.* These preliminary but encouraging results indicate this material might be suitable for the development of a more complete *in vivo* study including the application *in situ* of a static magnetic field.

## Conclusions

Overall, the *in vitro* results showed that the novel superparamagnetic FeHA MNPs not only did not reduce cell viability, but they enhance cell proliferation compared to HA particles controls already used in clinical application. Moreover, the positive effect of these nanoparticles was significantly increased when a SMF was applied.

In conclusion, the magnetic properties of the new FeHA phase open the door of regenerative medicine to a conceptually new family of biomimetic materials able to be biologically manipulated or “activated” *in situ* by means of an external magnetic field. Additionally, the wider field of theranostics may benefit of solutions represented by these completely biocompatible and biodegradable magnetic nano-carriers.

## Methods

### Synthesis of FeHA nanoparticles

FeHA nanoparticles were prepared according to the method described in our previous study [22]. Briefly, a basic suspension of calcium hydroxide Ca(OH)_2_ (Aldrich, 95 wt% pure, 50 g in 400 ml of H_2_O) was stirred and heated up to 40°C. FeCl_2_·4H_2_O (Aldrich, ≥ 99 wt% pure, 12.74 g in 75 ml of H_2_O) and FeCl_3_·6H_2_O (Aldrich, 97 wt% pure, 17.86 g in 75 ml of H_2_O) solutions were contemporarily added to the basic suspension as sources of Fe^2+^ and Fe^3+^ ions. The total amounts of iron ions with respect to calcium ions were adjusted so as to obtain: Fe/Ca = 20 mol%.

Soon after a phosphoric acid (Aldrich, 85 wt% pure, 44.40 g in 300 ml of H_2_O) solution was drop-wise added into a basic suspension of calcium hydroxide containing iron ions, over a period of 2 h, under constant heating and stirring.

The reaction products were kept in suspension by constant stirring and heating for 1 h and then left ageing for 24 h at room temperature without further stirring. The precipitate was separated from mother liquor by centrifugation, then washed with distilled water and centrifuged three times; finally it was freeze-dried and sieved at 150 μm.

For the *in vivo* test, FeHA nanopowder was processed in order to obtain a more stable granulate (400-600 μm). In detail, the powder was hydrated with distilled water and agglomerated. Agglomerates were dried at 40°C for 48 h and sieved in the range 400-600 μm. Glass vials containing 0.5 g of granulates were prepared and sterilized with 25 γ-ray. The control group consisted of a bone filler made of HA granulate of identical size (400-600 μm), already commercially available, and stabilized for one hour at 300°C (FinGranule, Finceramica Faenza Spa, Italy).

### FeHA nanoparticles characterization

FeHA phase composition was determined by X-ray powder diffraction (XRD), performed by a D8 Advance Diffractometer (Bruker, Karlsruhe, Germany) using CuKa radiation at 40 kV and 40 mA. XRD spectra were recorded in the 2 h range 10–60° or 15–120°, with a step size of 0.02 and a counting time of 1 s (corresponding to 185 s using a conventional detector). Quantitative evaluation of phase compositions and cell parameters was performed by full-profile Rietveld analysis of the XRD spectrum (TOPAS v4.2, Bruker AXS, Karlsruhe, Germany). Computer simulation of XRD patterns of FeHA powders based on structural models was carried out by the aid of the software Powder cell 2.4 (W. Krause & G. Nolze, 2000).

ICP-OES quantitative analysis made use of inductively coupled plasma – atomic emission spectrometry (ICP-AES: Liberty 200, Varian, Clayton South, Australia) to determine the overall content of Ca, P and Fe. Samples were previously prepared as follows: 20 mg of powder were dissolved in 2 ml of HNO3 (Aldrich, 65 wt% pure) and the solution volume was increased up to 100 ml with deionised water. The obtained values were expressed in terms of (Fe + Ca)/P mol, Ca/P mol and Fe/Ca molar %.

The analysis of powder morphology was carried out by high-resolution transmission electron microscopy (HRTEM) analyses, performed by a JEOL JEM 3010-UHR, operating at 300 kV.

Magnetic measurements were performed at higher field in a superconducting quantum interference device (SQUID) magnetometer from Quantum Design (San Diego, CA, USA), capable of operating from 1.8 to 350 K under a maximum applied magnetic field of H = 5 N A^-^1 m^-^1.

### Cell culture

Saos-2 Human Osteoblast-like cells purchased from Lonza (Italy) were cultured in Dulbecco Modified Eagle’s Medium (DMEM, PAA, Austria), containing penicillin/streptomycin (100 U/ml-100 μg/ml) supplemented with 10% fetal bovine serum and kept at 37°C in an atmosphere of 5% CO_2_. Cells were detached from culture flasks by trypsinization and centrifuged; cell number and viability were assessed with the trypan-blue dye exclusion test.

Saos-2 were plated at a density of 2.5 × 10^4^ cells/well in 24-well plates. 24 h after seeding, different concentrations of nanoparticles were added to the cell culture (2000 μg/ml; 1000 μg/ml; 500 μg/ml; 200 μg/ml). Nanoparticles were sonicated and vortexed before being added to the cells. Cells were incubated under standard conditions (37 °C, 5% CO_2_) with cell culture medium supplemented with 10 μg/ml ascorbic acid (Sigma) and 5 mM β-glycerophosphate (Sigma) for osteoblast activation, for 1, 3 and 7 days. Culture media was changed every other day. The experiments were conducted either with or without applying a static magnetic field (SMF) of 320 mT (MagnetoFACTOR-24, Chemicell, Germany) under the plates. All cell handling procedures were performed in a sterile laminar flow hood.

### Live/dead assay

Live/Dead Viability/Cytotoxicity assay kit for mammalian cells (Invitrogen) was performed according to manufacturer's instructions. Briefly, cells were washed with 1x PBS for 5 min and incubated with Calcein acetoxymethyl (Calcein AM) 2 μM plus Ethidium homodimer-1 (EthD-1) 4 μM for 15 min at 37°C in the dark. Cells were washed with 1x PBS for 5 min and images acquired by a confocal microscope Fluoview FV1000 (Olympus). The live and dead cells ratio was determined by quantifying the number of cells in 3 fields at the same magnification for each HA and FeHA nanoparticles concentration at each time point without magnetic field application. Two samples per time point were analysed for each group.

### Cell proliferation assay

Total DNA content was quantified using the Quanti-iT^TM^ PicoGreen dsDNA Assay Kit (Invitrogen) assay following the manufacturer’s suggested protocol. Seeded wells were washed with 1x PBS and then incubated with 1 ml 1x PBS with 0.1% (v/v) Triton-X for cell lysis. Collected cells were centrifuged at 11000 rpm for 1 min. 25 *μ*L of supernatant was added to 175  *μ*L of PicoGreen® reagent working solution in a 96-well plate. Fluorescence of the samples was measured with a microplate reader (Tecan, Research Triangle Park, NC) with excitation and emission wavelengths of 485 and 535 nm, respectively. A standard curve of fluorescence versus DNA concentration was created, from which the DNA concentration values for each sample were determined. The total number of cells in the sample was determined by converting the total DNA concentration to cell number using the conversion factor of 7.7 pg DNA/cell [31]. Five samples per condition per time point were analysed.

### Alkaline phosphatase assay

Cell Alkaline Phosphatase (AP) activity was quantified using an enzymatic assay based on the hydrolysis of *p*-nitrophenyl phosphate (pNP-PO_4_) to *p*-nitrophenol (pNP) [32]. Briefly, 25 μl of cell lysate, obtained as previously described (see the “cell proliferation assay” paragraph), was added to pNP-PO_4_ solution (Sigma-Aldrich) and allowed to react at 37°C. Absorbance was read at 0, 1, 2 and 3 min at λmax of 405 nm, using a microplate reader (Tecan, Research Triangle Park, NC) and AP activity calculated by cross-reference to a standard curve of nanomoles of p-nitrophenol liberated per cell. AP activity was normalized to total cellular content, as measured by the Picogreen assay. Five samples were analysed per condition per each time point.

### *In vivo* pilot experiment and histological analysis

The study was performed in accordance with EC guidelines (EC Council Directive 86/609, 1986) and the Italian legislation on animal experimentation (Decreto L. vo 116/92). The research protocol on animals has been approved by the Ethical Committee of Rizzoli Orthopaedic Institute and by the responsible public authorities. Six male rabbits (*Oryctolagus cuniculus,* Charles River, Lecco, Italy), 2.4 ± 0.2 kg body weight, were housed at a controlled temperature of 22 ± 1°C and relative humidity of 55 ± 5% in single boxes and fed a standard diet (Mucedola, Milano, Italy) with filtered tap water *ad libitum*. After quarantine of at least 10 days, the animals were fasted for 24 hours before surgery. The animals were subjected to surgery to implant the tested biomaterials at the distal femoral epiphysis under general anaesthesia and in aseptic conditions. After having shaved and disinfected the posterior legs, the animals underwent a lateral longitudinal incision of lateral femoral condyle. Femoral lateral condyle trabecular bone was cross-sectionally drilled at low speed and a profuse irrigation with cold sterile 0.9% NaCl solution was maintained throughout the process to prevent the risk of bone necrosis. A critical bone defect of 6.00 mm in diameter and 8.00 mm in depth was made in each lateral femoral condyle. Defects were filled with FeHA granules and with the HA granules in the contralateral condyle as a control group. For each defect, approximately 0.5 g granules, sterilized by 25 kGy γ-ray radiation, were used. Finally, the skin was sutured. General anaesthesia was induced by an intramuscular injection of 44 mg/kg ketamine (Imalgene 1000, Merial Italia S.p.A, Milan, Italy) and 3 mg/kg xylazine (Rompun, Bayer SpA, Milano, Italy) under assisted ventilation with O_2_/N_2_O (1/0.4 l/min) mixture and 2.5% isofluorane (Forane, Abbot SpA, Latina, Italy).

Post-operatively, antibiotics and analgesics were administered: 0.6 ml/kg flumequil (Flumexil, (FATRO SpA, Bologna, Italy) and 0.1 ml/kg/day metamizole sodium (Farmolisina, Ceva Vetem SpA, Monza-Brianza, Italy).

At 4 weeks after surgery, the animals were pharmacologically euthanized with intravenous administration of Tanax (Hoechst, Frankfurt am Main, Germany), under general anaesthesia. The operated bone segments were excised and stripped of soft tissue and the presence of haematomas, edema and inflammatory tissue reactions were macroscopically evaluated. The bone segments were fixed in 4% buffered paraformaldehyde for 24 hours, dehydrated in a graded series of alcohol and finally embedded in a methyl methacrylate resin (Merck Schuchardt OHG, Hohenbrunn, Germany). Using a saw microtome (Leica SP1600, Leica Microsystems Srl, Italy), three consecutive central sections to the major axis of the implant for each bone segment were cut (60 ± 20 μm) and polished (Struers Dap-7, Struers Tech A/S, Rodovre/Copenaghen, Denmark). Then, thinned sections (30 ± 10 μm) were stained with Toluidine Blue, Acid Fucsin and Fast Green.

### Statistical analysis

Results were expressed as MEAN±SEM plotted on graph (n = 5). Statistical analysis was performed using two-way ANOVA, followed by Bonferroni’s post-hoc test, for the analysis of magnetic field effect, time effect and magnetic field versus time effect. Analysis of differences between groups, for each time point, was performed by one-way ANOVA, followed by Tukey’s post-hoc test. All statistical analyses made were performed using GraphPad Prism software (version 5.0), with α=0.05.

## Competing interests

The authors declare that they have no competing interests.

## Authors’ contributions

Conceived and designed the experiments: SP, AT. Performed the experiments: SP, TD, GG. Analysed the data: SP, CC, MS, TD. Contributed materials: TD, MS, MM, CTH. Wrote the paper: SP, AT, CC. All authors read and approved the final manuscript.
